# Cohort Profile: IAVI’s HIV epidemiology and early infection cohort studies in Africa to support vaccine discovery

**DOI:** 10.1093/ije/dyaa100

**Published:** 2020-09-03

**Authors:** Matt A Price, William Kilembe, Eugene Ruzagira, Etienne Karita, Mubiana Inambao, Eduard J Sanders, Omu Anzala, Susan Allen, Vinodh A Edward, Pontiano Kaleebu, Patricia E Fast, Wasima Rida, Anatoli Kamali, Eric Hunter, Jianming Tang, Shabir Lakhi, Gaudensia Mutua, Linda Gail Bekker, Ggayi Abu-Baker, Amanda Tichacek, Paramesh Chetty, Mary H Latka, Pholo Maenetje, Heeran Makkan, Jonathan Hare, Freddie Kibengo, Fran Priddy, Elise Landais, Kundai Chinyenze, Jill Gilmour

**Affiliations:** 1 IAVI, New York, USA & Nairobi, Kenya; 2 Department of Epidemiology and Biostatistics, University of California at San Francisco, San Francisco, CA, USA; 3 Rwanda Zambia Emory HIV Research Group, Lusaka & Ndola, Zambia; Kigali, Rwanda; 4 Emory University, Atlanta, GA, USA; 5 Medical Research Council, Uganda Virus Research Institute, and London School of Hygiene and Tropical Medicine Uganda Research Unit (MULS), Entebbe & Masaka, Uganda; 6 Kenyan Medical Research Institute-Wellcome Trust, Kilifi, Kenya; 7 Nuffield Department of Clinical Medicine, Centre for Clinical Vaccinology and Tropical Medicine, University of Oxford, Headington, UK; 8 KAVI-Institute of Clinical Research, Nairobi, Kenya; 9 Department of Pathology and Laboratory Medicine, Emory University, Atlanta, GA, USA; 10 The Aurum Institute, Johannesburg and Rustenburg, South Africa; 11 School of Pathology, Faculty of Health Sciences, University of the Witwatersrand, Johannesburg, South Africa; 12 Advancing Care and Treatment for TB/HIV, A Collaborating Centre of the South African Medical Research Council, Cape Town, South Africa; 13 Department of Environmental Health Sciences, Yale School of Public Health, New Haven, CT, USA; 14 Pediatric Infectious Diseases, School of Medicine, Stanford University, Palo Alto, CA, USA; 15 Biostatistics Consultant, Arlington, VA, USA; 16 Department of Medicine, University of Alabama at Birmingham, Birmingham, AL, USA; 17 Desmond Tutu HIV Centre, University of Cape Town, Cape Town, South Africa; 18 Department of Epidemiology, Emory University, Atlanta, GA, USA; 19 IAVI Human Immunology Laboratory, Imperial College, London, UK; 20 IAVI Neutralizing Antibody Center, The Scripps Research Institute, La Jolla, CA, USA; 21 Department of Immunology and Microbiology, The Scripps Research Institute, La Jolla, CA, USA

## Why were the cohorts set up?

**Figure 1.**
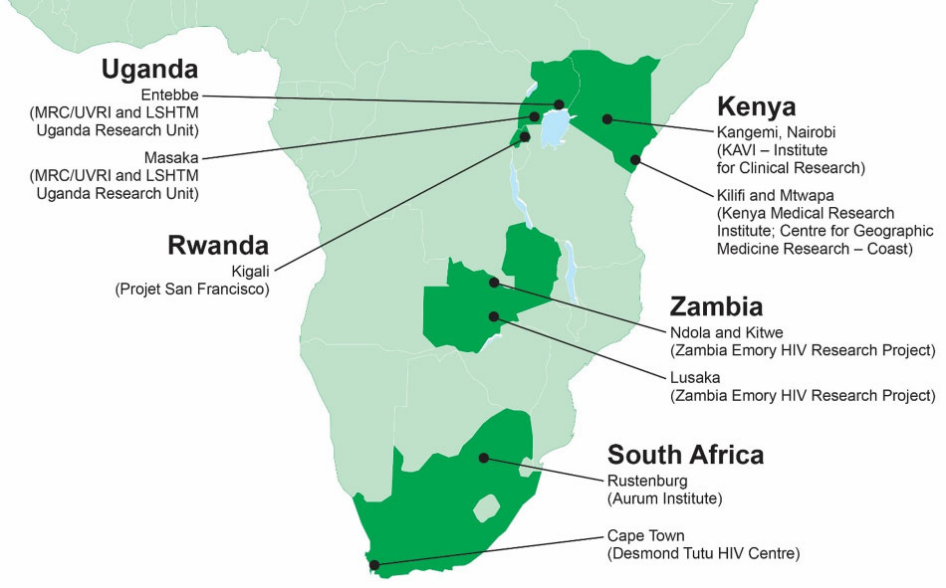
Participating clinical research centres.

In 2003, IAVI (formerly the International AIDS Vaccine Initiative) identified several gaps in HIV epidemiology and vaccine research. IAVI launched cohort studies to better understand at-risk populations, their suitability for clinical trial participation and their unmet needs for preventive services and products. Our goals included (i) improving our understanding of HIV incidence and volunteer retention among ‘key populations’ of at-risk persons suitable for participation in large-scale HIV prevention trials; (ii) identifying and addressing unmet needs for health care, counselling and prevention among these key populations; (iii) understanding host–virus interactions both shortly after virus acquisition and longer term; (iv) generating data and reagents from recently transmitted HIV to support new vaccine product discovery; and (v) understanding clinical outcomes of HIV disease in the African context to define clinical trial endpoints where antiretroviral therapy (ART) was not (yet) widely available, while building the clinical, laboratory and quality systems to support future trials. To this end, starting in 2004, IAVI established partnerships with nine experienced clinical research centres in east and southern Africa to enrol suitable volunteers (Figure 1). This manuscript includes data from two broad protocols that followed persons at risk of HIV acquisition (1) ‘A prospective, open cohort, observational feasibility study to determine HIV incidence in preparation for future preventive HIV vaccine clinical trials’ (IAVI Protocol B) and (2) ‘Heterosexual transmission of HIV in Africa’ [Emory Heterosexual Transmission (HT) study]. Between 2005 and 2009, varying by research centre, through to December 2011, they served as the source populations for a third cohort study on the natural history of HIV infection, ‘A prospective, observational, multi-center study to evaluate laboratory, clinical, immunologic and viral markers of disease progression in recently HIV-infected volunteers’ (IAVI Protocol C). Table 1 summarizes the start and stop dates for each site and each cohort. As part of participating in each respective cohort study, clinical and laboratory teams were trained in good clinical practices and good clinical laboratory practices, laboratories were accredited, and all assays were standardized and conducted under an external quality control programme.^1^ Study teams met annually, typically in Africa, to share experiences and results and for additional training.

**Table 1.** Overview of HIV epidemiology cohorts (Protocol B and Emory HT Study) and enrolment into the Early HIV Infection Cohort (Protocol C)

							Volunteers identified with incident HIV infection				
Recruitment city	Country	Protocol	Study population/ description^a^	Cohort size, *n*^b^	Earliest EDI	C Enrol stop date	Total, *n*	Enrolled into C, *n*	%	Others enrolled into C^c^	Total enrolled into C, *n*
Masaka	Uganda	B	Rural community based	1029	5 Sep 2005	30 Apr 2007	12	10	83.3	0	10
Masaka	Uganda	B	DC	1119	29 Jul 2006	31 Dec 2011	83	77	92.8	10	87
Entebbe	Uganda	B	DC	598	22 Feb 2006	17 Dec 2009	16	15	93.8	31	46
Kigali	Rwanda	HT	DC	1667	18 Feb 2005	15 Dec 2011	94	94	100.0	0	94
Lusaka	Zambia	HT	DC	1541	28 Jun 2005	15 Dec 2011	184	151	82.1	0	151
Ndola	Zambia	HT	DC	673	13 Oct 2005	15 Dec 2011	71	61	85.9	0	61
Kitwe	Zambia	HT	DC	243	14 Nov 2005	1 Dec 2008	25	22	88.0	0	22
Rustenburg	South Africa	B	At-risk men and women	599	2 May 2009	31 Dec 2011	22	21	95.5	1	22
Cape Town	South Africa	B	At-risk younger men and women	498	27 Oct 2006	27 Mar 2007	8	7	87.5	0	7
Kilifi	Kenya	B	MSM	508	15 Nov 2005	31 Dec 2011	77	67	87.0	1	68
Kilifi	Kenya	B	Heterosexual men	231	18 Sep 2006	31 Dec 2011	7	5	71.4	0	5
Kilifi	Kenya	B	At-risk women (FSW, other)	335	21 Mar 2006	31 Dec 2011	16	13	81.3	2	15
Kangemi	Kenya	B	MSM	301	9 Feb 2006	15 Sep 2010	19	17	89.5	4	21
Kangemi	Kenya	B	Heterosexual men	416	22 Jun 2009	15 Sep 2010	2	2	100.0	1	3
Kangemi	Kenya	B	At-risk women (FSW, other)	617	18 Sep 2006	15 Sep 2010	4	1	25.0	0	1
Total:				10 375			640	563	88.0	50	613

a DC, HIV discordant couples cohort; MSM, men who report sex with men; heterosexual men, men who report sex with women only; FSW, female sex workers.

b C source population: volunteers who contributed at least one follow-up visit while recruitment for Protocol C was active.

c Includes volunteers identified during serial voluntary counselling and testing visits (*n *= 31), prenatal visits (*n* = 1), discordant antibody test results (*n* = 7), participant in another epidemiology study with follow-up (*n* = 2) and p24-positive at screening for cohort study participation (*n* = 8).

## Who is in the cohorts?

To prepare for HIV vaccine efficacy trials, each team began outreach to and recruitment of populations suitable for large-scale prevention trials. Enrolment criteria varied by research centre and cohort, but generally included screening adults (typically 18–49 years old) for risk behaviour associated with an increased risk of HIV acquisition. Protocol B maintained a diverse range of higher-risk study volunteers (see below) whereas the HT study focused primarily on heterosexual transmission risk in stable, HIV-discordant couples (Table 1).

Starting in late 2004, the team in Masaka began recruiting volunteers in rural villages where previous research studies had found a higher HIV prevalence than the Ugandan national average.^2^ Annual HIV incidence was found to be low (1%), and the study team began to recruit the HIV-uninfected partner of HIV-discordant couples in 2006; this remained the source population for study volunteers at this recruitment centre for the duration of this study.^3^ Discordant-couple recruitment expanded to Entebbe in 2006.^4^ Also in 2004, IAVI began the support and expansion of discordant couple cohorts in Rwanda and Zambia, recruited through voluntary counselling and testing (VCT) sessions for couples.^5–7^ In Kilifi, recruitment began in 2005 with walk-in VCT attendees, then expanded to female sex workers and their clients. As the research centre became recognized as a site for non-judgmental care of sex workers, male sex workers began to attend outreach sessions and asked to be considered for enrolment; thus started the first prospective HIV epidemiology study among men who have sex with men (MSM) in Africa.^8^^,^^9^ In Nairobi, enrolment began in 2005 and volunteers included female sex workers, their clients, and MSM.^8^ In Cape Town, enrolment began in 2006, and because of high regional HIV prevalence and the generalized nature of the epidemic, recruitment included all persons reporting for VCT who reported any sexual activity; the Cape Town team was the only one to include adolescents, with an enrolment age range of 16–40 years old.^8^ Rustenburg initiated Protocol B in 2009, also focusing on men and women with more relaxed ‘risk’ criteria (any sexual activity). Many of these cohorts were active before recruitment for the Early HIV Infection Cohort began and continued after enrolment for that study ended. Additional details and a wider perspective on this work have been published elsewhere.^4^

Enrolment for Protocol C, the Early HIV Infection Cohort, began in February 2006, but included some volunteers whose HIV infection was diagnosed in 2005 and who were enrolled once the study started. These latter volunteers, diagnosed prior to the start of the Early HIV Infection Cohort, were invited to remain in their respective HIV incidence study where we provided CD4 T cell counts, counselling and appropriate care. We did not collect peripheral blood mononuclear cells (PBMCs) from those volunteers during these visits, as this was not permitted in the HIV epidemiology studies at that time. We did allow (with permission from the volunteers via the consent process) data and samples (in this case, frozen blood plasma only) from the incidence studies to ‘roll over’ into the Early HIV Infection Cohort Study once it began. Over 90% of Protocol C volunteers were identified via Protocol B and the HT Study, with the remainder either identified at the time of screening for Protocol B by detectable p24 antigen in the absence of HIV antibodies (suggesting incident HIV infection) or through other sources (Table 1, see also^10^). Suspected transmitting partners were also invited to enrol for a single study visit and 406 partners were enrolled, primarily from the HIV-discordant couple cohorts. In 2006, ART programmes in east and southern Africa were in their infancy, and ART was typically initiated when CD4 T cell counts were ≤200 cells/mm^3^. These programmes evolved considerably over the course of the Early HIV Infection Cohort, with 17 major guideline changes across five countries from 2005 to 2011. Volunteers’ health was monitored closely and ART was initiated per guidelines that existed at the time.

## How often have they been followed up?

Volunteers in Protocol B and the HT Study were typically followed quarterly, with a subset deemed at higher risk for HIV acquisition followed monthly for more prevention counselling and HIV testing to detect incident HIV soon after transmission (see ‘What has been measured?’ below). As volunteers were diagnosed with HIV infection they were invited to enrol in the Early HIV Infection Cohort and their follow-up schedule was determined based on their estimated date of HIV infection (EDI). The EDI was defined as the midpoint between the date of the last negative and first positive test in the case of detection by HIV antibody assay, 14 days prior to the test date in the case of detection by p24 assay only, or 10 days prior to the test date for those volunteers with a polymerase chain reaction (PCR)-positive result prior to antibody or p24 detection. If a volunteer could identify an obvious exposure event, the date of this event could be adopted as the EDI at the discretion of the research team. Once enrolled into the Early HIV Infection Cohort, volunteers were followed monthly for the first 3 months after EDI, quarterly through 2 years post-EDI, and every 6 months thereafter. Of the Early HIV Infection Cohort volunteers, 112 (18%) volunteers were identified very soon after acquiring HIV infection (typically prior to full HIV antibody seroconversion) and were invited into a different visit schedule for the first 3 months: weekly follow-up visits with PBMC collection in the first month following enrolment, then every 2 weeks for the second and third month. Subsequently, their follow-up schedule matched the other Early HIV Infection Cohort volunteers.

## What has been measured?

Each HIV epidemiology cohort study measured basic demographics at enrolment, with risk behaviour assessed at enrolment and at each quarterly visit thereafter. Medical history and physical examination were performed at baseline, genital examination was performed either at baseline or as indicated if sexually transmitted infection (STI) was suspected. HIV testing was done quarterly and followed the national guidelines; typically, they recommended two rapid tests followed by a tiebreaker if needed. A p24 antigen test was also performed to detect HIV infection prior to seroconversion. These data are summarized in Table 2. Plasma was stored from each visit. If incident HIV infection was detected, additional sampling and tests were done, including PCR testing of the preceding study visit sample to detect HIV infection prior to seroconversion (Table 2). Newly transmitted HIV was subtyped by sequencing the *pol* region of the genome and transmitted antiretroviral mutations were assessed, as described.^11^ Risk was evaluated at month 12 in Protocol B only, and volunteers who were no longer eligible in terms of behaviour risk were taken off study. Volunteers in the HT study continued follow-up while in a sexual relationship with an HIV-positive person. Once enrolled into the Early HIV Infection Cohort, larger volumes of blood were collected to allow processing and storage of plasma and PBMCs, HIV viral load testing, and CD4 and CD8 T cell counts. Human leukocyte antigen system (HLA) characterization was done for all Early HIV Infection Cohort volunteers, as described elsewhere.^12^ Data on medication history, including antiretroviral drugs, was also collected. As these volunteers went on antiretroviral therapy, they were followed to assure they were medically stable and enrolled in a treatment programme, then taken off study.

**Table 2.** Data collected in Protocol B, the Emory H) Study and Protocol C

	Baseline demographics	Medical history, physical exam	Risk assessment	Risk behaviour data	HIV VCT	HIV testing	Other testing
Protocol B	Age, sex, education, race, tribe where available (e.g. recording data on tribe is not legal in Rwanda), country of birth, education, religion, marital status (including polygamous marriage), duration of time in current domicile	Baseline and quarterly. Includes external genital examination at baseline, symptoms-directed examinations at quarterly follow up	Quarterly^a^ except at Masaka where it was done every 6 months	Alcohol and drug use, self-reported STI symptoms, number of sex partners, number who were new, condom frequency, knowledge of partners' HIV status, commercial sex, forced sex/rape, group sex, anal sex	Quarterly^a^	Two antibody rapid tests, with third tiebreaker (rapid or ELISA^b^), p24 antigen ELISA	Syphilis annually, symptoms-directed STI testing, urine pregnancy testing quarterly. Safety labs^c^, plasma and PBMC collection if incident HIV detected
HT Study	Age, sex, literacy (local language and English), occupation, income, length of stay in city, religion, tribe, alcoholic history, polygamy (men) and type of marriage (men)	Baseline and quarterly. Genital examination at baseline, symptom- directed and at annual follow up	Quarterly^a^	Frequency of sex with and without condom with HIV+ partner, frequency of sex with additional partners (if any)	Quarterly^a^	Two antibody rapid tests, with third tiebreaker (rapid or ELISA), p24 antigen ELISA	Hematocrit and syphilis quarterly. Symptoms-directed STI testing. Safety labs, plasma and PBMC collection if incident HIV detected
Protocol C	Age, sex, education, race, tribe where available, country of birth, education, marital status (including polygamous marriage)	Baseline and quarterly. Includes external genital examination at baseline, symptom-directed examinations and concomitant medications (including ARVs^d^) at quarterly follow up	Baseline only	High-risk activities in 3 months prior to enrolment, suspected route of exposure for those volunteers who could recall a specific exposure event	Not applicable	Confirmation of p24 antigen status and/or antibody seroconversion, viral load PCR at every visit	Syphilis annually, symptom-directed STI testing, urine pregnancy testing quarterly. Safety labs, plasma and PBMC collection. HLA characterization

a Monthly in a subset of volunteers deemed at higher risk.

b ELISA, enzyme-linked immunosorbent assay.

c Safety labs include hematology (Complete Blood Count (CBC), differential, platelets), serum chemistries (alanine transaminase (ALT), creatinine (Cr)), CD4 and CD8 T cell counts.

d ARV, antiretroviral drugs.

## What has been found? Key findings and publications

To date, data and samples from these cohorts have contributed to over 220 peer reviewed manuscripts. Highlights of this research include the following.

- **HIV epidemiology**: Even in the context of regular counselling and testing (and well before the era of widespread ART availability, pre-exposure prophylaxis and test and treat programmes) HIV incidence remained high. In these cohorts, we observed annual HIV incidences ranging from ∼1% in rural Ugandans, 4% in Ugandan discordant couples, 3% in Rwandan discordant couples, 8% in Zambian discordant couples, 6–7% in Kenyan MSM, 9–10% in South African women and 2–3% in Kenyan female sex workers.^2–4^,8 HIV incidence tended to vary by sex, time on study, and sometimes by calendar year though there was considerable heterogeneity across cohorts.^4^ Our Kenyan cohort of MSM was the first of its kind in Africa and highlighted many similar risk factors for HIV acquisition to non-African MSM, including unprotected sex, group sex, other STIs (e.g. gonorrhea) and receptive anal intercourse.^13^ Working with these men, we have created training modules for health care workers to improve health care delivery and reduce prejudice.^14^^,^^15^ Additional work included some of the first published reports on transmitted drug resistance mutations in Africa,^11^ characterizing pregnancy outcomes in women with HIV infection,^16^ describing a novel HLA type associated with favourable clinical outcomes, B*44 (12), and confirmation of other HLA types and other factors in disease progression.^17^^,^^18^ Ground-breaking work with HIV discordant couples has reinforced the importance of couples’ VCT in lowering HIV incidence,^19–21^ leading the World Health Organization (WHO) to recommend couples’ counselling wherever VCT is available.^22^
- **Clinical course of HIV infection**: When the Early HIV Infection Cohort began, national ART programmes in the study countries were just beginning and criteria for when to start varied. Volunteers were followed closely, their CD4 T cell counts and viral loads monitored at every visit and they were referred for treatment according to national guidelines current at the time. The cohorts included regions with very different epidemic dynamics and viral diversity, allowing comparison of clinical outcomes across infecting HIV-1 subtype. We observed that HIV disease progression measured by three endpoints varied by subtype, with C-infected volunteers tending to progress to (i) AIDS, (ii) viral load ≥100 000 copies/mL and (iii) CD4 T cell count ≤350 cells/µl faster than those infected with subtype A.^23^ Because we also enrolled volunteers with incident HIV infection, typically within 1–2 months of their EDI, we were able to see patterns in acute retroviral syndrome; those infected with subtype A appeared to have worse symptoms shortly after infection, with greater report of headache, lymphadenopathy, fever and other symptoms.^24^ T cell decline in very early infection was much more pronounced than expected, with a majority of volunteers falling below 500 cells/µL (the WHO-recommended threshold to start treatment in 2013) within 6 months of acquiring HIV infection.^25^ We also observed that ∼5% of volunteers appeared to control the virus, keeping viral load ≤2000 copies/mL. This too varied by infecting subtype; those with subtype A were more likely to control the virus compared with those with subtype C.^10^
- **HIV transmission**: Enrolment of suspected transmitting partners allowed for in-depth analysis of events around the time of HIV transmission. We observed that 67–100% of suspected transmitting partners were truly the index case by comparing sequence between partners, and that this varied by study site.^4^ Infection is typically established by a single genetic variant from the HIV swarm in the index case; we observed selection bias towards more fit viral variants establishing new infection, and that this bias was increased in men compared with women, suggesting a more permissive transmission environment in the female genital tract.^26^ We also observed that these bottleneck events were not as pronounced when inflammation and/or STIs were present, which presumably compromised the mucosal barrier to viral entry allowing transmission of greater numbers and/or diversity of variants.^26^^,^^27^ Pre-adapted HIV, that is, transmitted HIV that came from someone with a similar HLA profile to the recipient’s, was also found to be associated with more negative clinical outcomes—this preadaptation likely grants recently transmitted HIV a level of invisibility to the new host’s immune system to which it may, in part, have already been adapted through prior immune escape.^28^^,^^29^
- **HIV virology**: *Pol* sequence was determined at an early timepoint for all volunteers, to estimate the HIV subtype.^12^ Efforts to generate full-length sequence and infectious molecular clones from very early samples (typically within 2 months of the EDI) are underway to further define the extent of genetic recombination between regions and subtypes. Viral replicative capacity, as defined by cloning the virus’ gag sequence into a replication-competent viral backbone (MJ4 and NL-43) and quantifying subsequent viral replication via an *in vitro* cell culture assay,^30^ was found to correlate with immune decline, independent of viral load and HLA type; viruses with high replicative capacity were also found to more readily infect memory T cells, suggesting a more efficient seeding of the latent viral reservoir.^31^
- **Neutralizing antibodies to HIV infection**: Understanding how the body produces neutralizing antibodies to HIV is currently an exciting topic for HIV vaccine design. Although individuals infected with HIV make neutralizing antibodies to the infecting virus, it rapidly escapes through mutations.^32^^,^^33^ A goal, therefore, of the Early HIV Infection Cohort Study was to characterize the frequency and development of broad and potent neutralizing antibodies to HIV. In this cohort, we observed that ∼15% of volunteers developed broadly neutralizing antibodies to HIV, typically between 2 and 4 years post-infection. Higher viral loads, lower CD4 T cell counts, HLA A*03 allele and infection with subtype C HIV were all independently associated with the development of neutralizing antibodies.^34^ In-depth characterization of the process over time by which these antibodies are developed provides guidance for immunogen design for an HIV vaccine to elicit neutralizing antibodies against a wide array of epitopes.^35–37^
- **Contributing to larger-scale a**na**lyses**: Samples and data from these studies have contributed to larger work, including the development of assays to estimate HIV incidence from prevalent samples,^38^^,^^39^ the creation of a repository of reagents of recently transmitted HIV to help researchers and assay developers,^40^ and to answer questions about HIV and other infectious diseases in the context of European and global cohorts, including issues of host genetics and viral control, differences between European and African cohorts, and co-infections such as Hepatitis C.^41–45^ The samples and data are being used by many African scientists and post-doctorate investigators to develop translational research capabilities, training opportunities and to strengthen north–south and south–south partnerships within Africa.

## What are the main strengths and weaknesses?

These cohorts were set up to both address questions of HIV epidemiology and volunteer retention, as well as recruit volunteers for Protocol C, our early infection cohort. We enrolled a diverse set of cohorts across a diverse epidemic: HIV subtypes included A, C, D and many recombinant viruses. In part because of our strong relationships with each respective community, we had great success enrolling volunteers with incident HIV—∼90% of those diagnosed in Protocol B and the HT study enrolled into Protocol C (Table 1). Retention was high in the Early HIV Infection Cohort, with an annual attrition, on average, of ∼5%. Early timepoints and the collection of PBMCs has enabled us to answer questions related to the events early in infection, from viral dynamics to host immunology. However, because the Early HIV Infection Cohort was set up to answer questions relevant for HIV vaccine trials in an era prior to test and treat (e.g. clinical outcomes that might be amenable to therapeutic vaccines), we did not systematically follow volunteers once they started ART; questions about ART treatment programmes, ART effectiveness and their outcomes may not be well suited to this cohort. Additionally, as costs would have been prohibitive, we did not collect pre-infection PBMCs from all volunteers enrolled into the HIV incidence cohorts and thus have no details on immune status prior to HIV acquisition.

## Can I get hold of the data? Where can I find out more?

We are committed to the tenets of Open Data and actively encourage investigators to reach out to us for more information, particularly African scientists from these regions. Data are currently available online and samples can be requested. These data are managed at the IAVI Dataspace, found at https://dataspace.iavi.org/. Data may also be found online with each respective publication that requires adherence to Open Data policies, and HIV sequence data have been submitted to GenBank. More information about IAVI’s epidemiology program and how to obtain data and samples from these studies can be found online at https://www.iavi.org/our-work/iavi-dataspace.

## Collaboration and data access

We welcome invitations for collaboration. IAVI maintains an online DataSpace with details on how to contact us and what data and samples are available (https://dataspace.iavi.org/).

## Funding

This work (i.e. Protocol B, HT Study and Protocol C) was supported by the United States Agency for International Development (USAID). The following grants also supported the HT Study in Rwanda and Zambia: NIH-NIAID P30 AI050409, NIH-FIC TW001042, NIH-NIAID R01 AI040951, NIH-NIMH R01 MH066767, NIH-NICHD R01 HD040125, NIH-NIAID R01 AI064060, and NIH-NIAID R37 AI51231. Some of the Early HIV Infection Cohort (Protocol C) work done at Emory was supported by the Yerkes National Primate Research Center base grant through the Office of Research Infrastructure Programs (OD P51OD11132). HIV neutralizing antibody work was funded in part by the National Institute Of Allergy And Infectious Diseases (grant number U19AI090970) as well as Bill and Melinda Gates Foundation Collaboration for AIDS Vaccine Discovery grants numbered OPP1084519 (2013–2018), OPP1196345 (2019–2021) and OPP1115782 (2015–2019).
